# Design of an Evolutionary Approach for Intrusion Detection

**DOI:** 10.1155/2013/962185

**Published:** 2013-11-25

**Authors:** Gulshan Kumar, Krishan Kumar

**Affiliations:** Shaheed Bhagat Singh State Technical Campus, Ferozepur, Punjab 152004, India

## Abstract

A novel evolutionary approach is proposed for effective intrusion detection based on benchmark datasets. The proposed approach can generate a pool of noninferior individual solutions and ensemble solutions thereof. The generated ensembles can be used to detect the intrusions accurately. For intrusion detection problem, the proposed approach could consider conflicting objectives simultaneously like detection rate of each attack class, error rate, accuracy, diversity, and so forth. The proposed approach can generate a pool of noninferior solutions and ensembles thereof having optimized trade-offs values of multiple conflicting objectives. In this paper, a three-phase, approach is proposed to generate solutions to a simple chromosome design in the first phase. In the first phase, a Pareto front of noninferior individual solutions is approximated. In the second phase of the proposed approach, the entire solution set is further refined to determine effective ensemble solutions considering solution interaction. In this phase, another improved Pareto front of ensemble solutions over that of individual solutions is approximated. The ensemble solutions in improved Pareto front reported improved detection results based on benchmark datasets for intrusion detection. In the third phase, a combination method like majority voting method is used to fuse the predictions of individual solutions for determining prediction of ensemble solution. Benchmark datasets, namely, KDD cup 1999 and ISCX 2012 dataset, are used to demonstrate and validate the performance of the proposed approach for intrusion detection. The proposed approach can discover individual solutions and ensemble solutions thereof with a good support and a detection rate from benchmark datasets (in comparison with well-known ensemble methods like bagging and boosting). In addition, the proposed approach is a generalized classification approach that is applicable to the problem of any field having multiple conflicting objectives, and a dataset can be represented in the form of labelled instances in terms of its features.

## 1. Introduction

The industry faces the challenges of fast changing trends of attacking the internet resources, inability of conventional techniques to protect the internet resources from a variety of attacks, and biases of individual techniques towards specific attack class(es). Developing effecting techniques is necessary for securing valuable internet resources from attacks. Nowadays, conventional protection techniques such as firewalls, user authentication, data encryption, avoiding programming errors, and other simple boundary devices are used as the first line of defense for security of the systems. Some attacks are prevented by the first line of defense whereas some bypass them. Such attacks must be detected as soon as possible so that damage may be minimized and appropriate corrective measures may be taken. Several techniques from different disciplines are being employed for the accurate intrusion detection systems (IDSs). Detection rate (DR) and false positive rate (FPR) are two key indicators to evaluate the capability of an IDS. Many efforts are being done to improve DR and FPR of the IDSs [1]. In the beginning, the research focus was to rule based IDSs and statistical IDSs. But, with large data sets, the results of these IDSs become unsatisfactory. Thereafter, a lot of AI based techniques have been introduced to solve the problem due to their advantages over the other techniques [2, 3]. The AI based techniques have reported certain improvements in the results to detect the intrusions. Many researchers analyzed various AI based techniques empirically and compared their performance for detection of intrusions. Findings of representative empirical comparative analysis are as follows. Most of the existing techniques strive to obtain a single solution that lacks classification trade-offs [4]; low detection accuracy, high false alarm rate; no single technique is capable enough to detect all classes of attacks to an acceptable level of false alarm rate and detection accuracy [2, 5]; some of the existing techniques fall into local minima. For global minima, these techniques are computationally expensive; the existing techniques are not capable of modeling correct hypothesis space of the problem [6]; some existing techniques are unstable in nature such as neural networks showing different results with different initialization due to the randomness inherent in the training procedure; different techniques trained on the same data may not only differ in their global performances, but they may show strong local differences also; Each technique may have its own region in the feature space where it performs the best [7]; delay in the detection of intrusions due to the processing of a large size of high dimensional data [3, 8]; NB, MLP, and SVM techniques are found to be the most promising in detecting the intrusions effectively [9]. It is also noticed from the literature of AI based techniques that most of the existing intrusion detection techniques report poor results in terms of DR and FPR towards some specific attack class(es). Even, artificial neural networks (ANNs), naive bayes (NB), and decision trees (DT) have been popularly applied to intrusion detection (ID), but these techniques have provided poor results, particularly towards the minor attack class(es) [10, 11]. The poor results may be due to an imbalance of instances of a specific class(es) or the inability of techniques to represent a correct hypothesis of the problem based on available training data.

In order to improve the low DR and high FPR, the focus of the current research community in the field of intrusion detection (ID) is on ensemble based techniques, because there is a claim in the literature that ensemble based techniques generally outperform the best individual techniques. Moreover, several theoretical and empirical reasons including statistical, representational, and computational reasons exist that also advocate the use of ensemble based techniques over the single techniques [12].

This paper is devoted to develop an evolutionary approach for intrusion detection that generates a pool of noninferior individuals' solutions and combines them to generate ensemble solutions for improved detection results. The pool of solutions provides classification trade-offs to the user. Out of pool of solutions, the user can select an ideal solution as per application-specific requirements.

The rest of the paper is organized as follows.  Section 2 presents the related work and identifies the research gaps in the field. A novel evolutionary approach for effective intrusion detection is proposed in Section 3. This section also gives details of experiments including a brief description of GA, NB, benchmark data sets, performance metrics followed by experimental setup, and the results of the proposed approach using NB as a base classifier. Finally, the concluding remarks along with the scope for future work are listed at the end of this paper in Section 4.

## 2. The Literature Review

Ensemble techniques/classifiers have been recently applied to overcome the limitations of a single classifier system in different fields [12–14]. Such attention is encouraged by the theoretical [12] and experimental [15] studies, which illustrate that ensembles can improve the results of traditional single classifiers. In general, an ensemble construction of base classifiers involves generating a diverse pool of base classifiers [16], selecting an accurate and diverse subset of classifiers [17], and then combining their outputs [13]. These activities correspond to ensemble generation, ensemble selection, and ensemble integration phases of the ensemble learning process [18]. Most of the existing ensemble classifiers aim at maximizing the overall detection accuracy by employing multiple classifiers. The generalizations made concerning ensemble classifiers are predominantly suitable in the field of ID. As Axelsson [19] notes, “In reality there are many different types of intrusions, and different detectors are needed to detect them.” Use of multiple classifiers is supported by the statement that if one classifier fails to detect an attack, then another should detect it [20]. However, to create an efficient ensemble, we are still facing numerous difficulties: how can we generate diverse base classifiers? Then, once these base classifiers have been generated, should we use all of them or should we select a subgroup of them? If we decide to select a subgroup, how do we go about it? Then, once the subgroup has been selected, how can we combine the outputs of these classifiers?

Previous studies in the field of intrusion detection have attempted various techniques to generate effective ensembles such as bagging, boosting, and random subspace. Giacinto and Roli [21] proposed a multiclassifier based system of neural networks (NNs). The different neural networks were trained using different features of KDD cup 1999 dataset. They concluded that a multistrategy combination technique like belief function outperforms other representative techniques. A multiclassifier system of NNs was also advocated by Sabhnani and Serpen [22]. The authors reported improved results over single techniques. Chebrolu et al. [23] and Abraham and Thomas [24] used weighted voting to compute the output of an ensemble of CART and BN and reported improved results for intrusion detection. Perdisci et al. [25] proposed a clustering based fusion method that reduces the volume of alarms produced by the IDS. The reduced alarms provide a concise high level description of attacks to the system administrator. The proposed method uses the correlation between alarms and meta alarms to reduce the volume of alarms of the IDSs. A hierarchical hybrid system was also proposed by Xiang et al. [26]. But, the proposed system leads to high false positive rate. Chen et al. [27] used the different features of dataset to generate ensemble solutions based on evolutionary algorithms. Toosi and Kahani [28] proposed a neurofuzzy classifier to classify instances of KDD cup 1999 dataset into five classes. But, a great time consuming is a big problem. Hu and Damper [29] proposed an adaBoosting ensemble method that uses different features to generate a diverse set of classifiers. No doubt, the proposed method reported improved performance but it suffers from the limitation of incremental learning. It requires continuous retraining for a changing environment. Zainal et al. [30] proposed a heterogeneous ensemble of different classifiers and used weighted voting method for combining their predictions. Wang et al. [31] proposed an approach based on NN and fuzzy clustering. Fuzzy clustering helps to generate homogeneous training subsets from heterogeneous training datasets which are further used to train NN models. They reported improved performance in terms of detection precision and stability. Clustering based hybrid system was also advocated by Muda et al. [32] for intrusion detection. The system was unable to detect the intrusions of U2R and R2L attack classes. Khreich et al. [33] proposed an iterative Boolean combination (IBC) technique for efficient fusion of the responses from any crisp or soft detector trained on fixed-size datasets in the ROC space. However, IBC does not allow to efficiently adapt a fusion function over time when new data become available, since it requires a fixed number of classifiers. The IBC technique was further improved as incremental Boolean combination (incrBC) by the authors [14]. The incrBC is a ROC-based system to efficiently adapt ensemble of HMM (EoHMMs) over time, from new training data, according to a learn-and-combine approach without multiple iterations. Govindarajan and Chandrasekaran [34] suggested a hybrid architecture of NNs for intrusion detection. They used the weighted voting method to compute the final prediction of the system.

However, the models developed based on these techniques attempted to obtain a single solution. They have a lack in providing classification trade-offs for application specific requirements. Most of the models provided biased results towards specific attack class(es).

In contrast, evolutionary algorithm seems to be well suited for the solution of multiobjective optimization (MOO) problems mainly due to their inherent characteristics concerning the population set based exploration of the search space of a given problem [35]. Out of many evolutionary algorithms, multiobjective genetic algorithm (GA) is the most widely used technique in data mining and knowledge discovery [36]. Applying GA is valuable for its robustness in performing a global search in search space compared with other representative techniques. Several researchers employed single and multiple objective genetic algorithms for finding a set of noninferior solutions for the problem of ID. Such initiative was carried by Parrott et al. [37] by suggesting an evaluation function which was later known as Parrot function. They proposed to use accuracy of each target class as a separate objective in their evaluation function for multiobjective GA. Here, accuracy of each class refers to correctly classified instances of that class. The Parrot function was further adopted by Ahmadian et al. [38, 39] to generate an ensemble of base classifiers. The generation of the ensemble was completed in two stages using modified NSGA-II [40]. In the first stage, a set of base classifiers was generated. Second stage optimized the combination of base classifiers using a fixed combining method. Both of these methods differ in their function evaluation. The former study proposed to optimize the classifiers by minimizing the aggregated error of each class and maximizing diversity among them. Since the error on each class is not treated as separate objective; this is similar to a general error measure such as mean square error MSE, which has the same issues as the implementation of Parrot function, being biased towards the major class(es). In the second phase of the approach proposed by Ahmadian et al. [38, 39], the objectives are to minimize the size of the ensemble and maximize the accuracy. Consequently, the drawback of their approach is to create a single best solution based on general performance metrics. The same concept was further extended by Egen [4] by conducting similar experiments with different evaluation functions for creating an ensemble of ANNs as base classifiers in the presence of imbalanced datasets using NSGA-II. He used 3-class classification by using ANNs and multiobjective GA. He proved that multiobjective GA based approach is an effective way to train the ANN which works well for minority attack classes in imbalanced datasets. He proposed two-phase process for intrusion detection. In the first phase, he generated a set of base classifiers of ANNs by optimizing their weights assuming a fixed number of hidden layers and the number of neurons per hidden layer in ANN. The second phase generates improved nondominated front of ensemble solutions based upon base ANN solutions optimized in phase 1. However, the performance of NSGA-II degrades for the real world problems having more than three objectives and large population [41].

## 3. Evolutionary Approach for Intrusion Detection

A novel evolutionary approach based on multiobjective GA for intrusion detection is proposed. The concept of two-tier fitness assignment mechanism consisting of domination rank and the diversity measure of solutions (as proposed by Tiwari [42]) is used to improve the solutions from benchmark datasets. Generally, the intrusion detection problem encounters a trade-off between multiple conflicting criteria such as the detection rate of attack classes, accuracy, and diversity. An exact solution to such multiobjective problem at which decision variables satisfy the related conditions and all objectives have attained corresponding optimal values may not even exist [35]. Usually, there is no single solution to a multiobjective problem but rather a set of optimal solutions called Pareto optimal solutions. All the solutions in this set are noninferior to any other solutions when all objectives are considered. In fact, evolutionary algorithms especially multiobjective GA attempt to optimize each individual objective to a maximum extent. Thus, considering the multiple criteria of the intrusion detection problem, GAs can be used in two ways. The first way to solve a multiobjective problem is to convert multiple objectives into a single objective [43]. The single objective is further optimized by GA to produce a single solution. Generally, prior knowledge about the problem or some heuristics guides the GA to produce a single solution. By changing the parameters of the algorithm and executing the algorithm repeatedly, more solutions can be produced. This approach has several limitations for multiobjective optimization problems. The second way to solve multiobjective optimization problems is by using GA producing a set of noninferior solutions. This set of noninferior solutions represents trade-offs between multiple criteria which are identified as a Pareto optimum front [4, 44]. By incorporating domain knowledge, the user can select a desired solution. Here, GA has produced a set of solutions in Pareto front in a single run without incorporating any domain knowledge or any other heuristics about the problem. Some of the important researches in developing multiobjective GAs are a strength Pareto evolutionary algorithm (SPEA2) [45], Pareto-envelope based selection algorithm (PESA-II) [46], nondominated sorting genetic algorithm (NSGA-II) [47], archive based microgenetic algorithm 2 [48], and many more. A comprehensive review of various multiobjective GAs can be further referred to in [43, 47, 49].

The proposed approach is developed with particular attention to enhance the detection rate of majority as well as minority attack class(es). A chromosome encoding scheme is proposed to represent the individual classifiers. Furthermore, the proposed approach is used to find an improved Pareto front consisting of ensemble solutions. The multiobjective GA used in this paper is archive based microgenetic algorithm 2 (AMGA2) [48], which is an effective algorithm for finding optimal trade-offs for multiple criteria. AMGA2 is a generational algorithm that works with a very small population size and maintains a large external archive of good solutions obtained. Using an external archive that stores a large number of solutions provides useful information about the search space as well as tends to generate a large number of Pareto points at the end of the simulation. At every iteration, a small number of solutions are created using the genetic variation operators. The newly created solutions are then used to update the archive. The strategy used to update the archive relies on the domination level and the diversity of the solutions and the current size of the archive and is based on the nondominated sorting concept borrowed from NSGA-II [40]. This process is repeated until the allowed number of function evaluations is exhausted. We used differential evolution (DE) operator as crossover operator for mating the population. DE has the advantage of not requiring a distribution index, and it is self-adaptive in that the step size is automatically adjusted depending upon the distribution of the solutions in the search space. After mating the population with crossover operator, it is followed by mutation operator. The modified polynomial mutation operator is used to mutate the offspring's solutions.

### 3.1. The Proposed Evolutionary Approach

**Figure 1:**
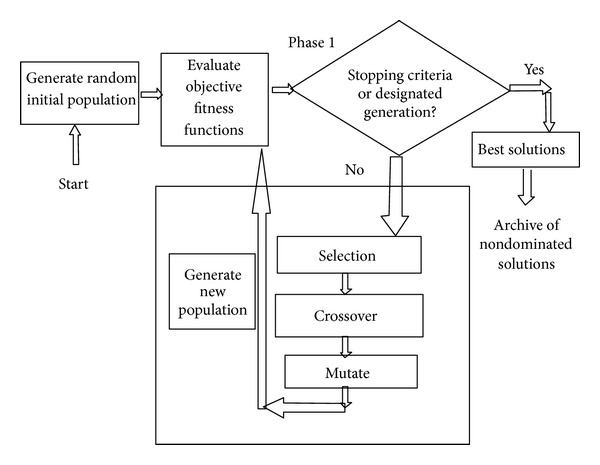
Phase 1 of the proposed approach.

**Figure 2:**
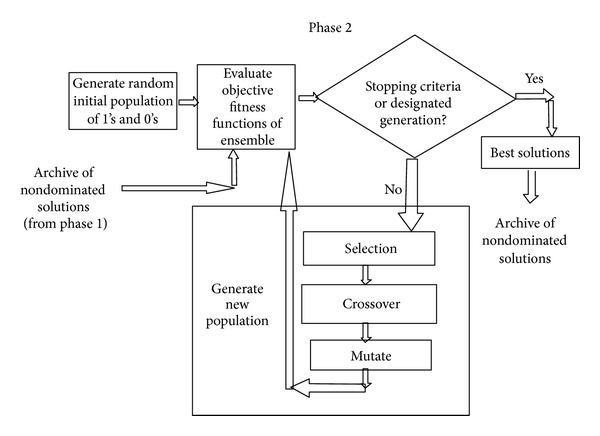
Phase 2 of the proposed approach.

**Figure 3:**
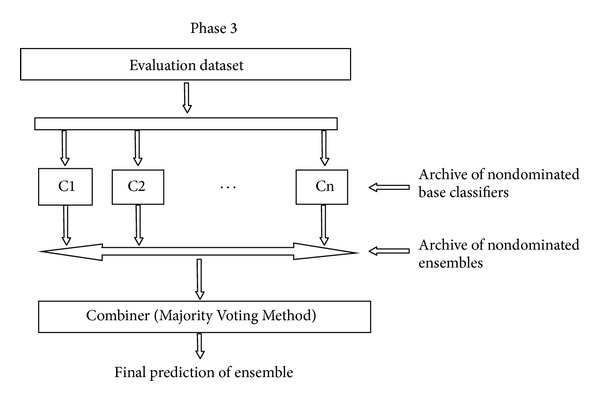
Phase 3 of the proposed approach.

This section describes the proposed approach based on multiobjective GA to create a set of base classifiers and ensembles thereof. The proposed approach follows overproduce and choose approach. It focuses on the generation of a large number of base classifiers and later on choosing the subset of the most diverse base classifiers to generate ensembles. The proposed approach is a three-phased technique as described in subsequent paragraphs. Phases 1 and 2 are multiobjective in nature and use multiobjective GA to generate a set of base classifiers and ensembles thereof, respectively. The set of base classifiers and their ensembles exhibit classification trade-offs for the user. Phase 1 evolves a set of individual solutions to formulate diverse base classifiers using multiobjective GA. The diversity among base classifiers is maintained implicitly. The detection rate for each class is treated as a separate objective. Here, the multiobjective GA is real coded and uses crossover and mutation operators and an elitist replacement strategy. This phase of multiobjective GA is able to find the optimal Pareto front of nondominated solutions (depicted in Figure 1). These solutions formulate the base classifiers as candidate solutions for the ensemble generation in phase 2. In phase 1, AMGA2 is real coded using its crossover and mutation operators. The values in chromosome and its size depend upon the type of base classifier and corresponding encoding scheme. The output of phase 1 is a set of optimized real values for classifiers that formulate the base classifiers of ensembles. The population size is equal to the number of desired solutions input by the user. Phase 2 generates another improved approximation of optimal Pareto front consisting of a set of nondominated ensembles based on a pool of nondominated solutions as base classifiers (output of phase 1) which also exhibit classification trade-offs (depicted in Figure 2). It takes input in the form of archive of nondominated solutions produced by phase 1 that formulates the base classifiers of the ensembles. The phase evolves ensembles by combining the Pareto front of nondominated solutions instead of the entire population like other studies [50]. The detection rate for each class is treated as a separate objective. Here, we are interested in those solutions which are noninferior and exhibit classification trade-offs. The predictions of the base classifiers are combined using the majority voting method. In case of a tie, the winner is randomly chosen. The multiobjective GA method discussed in phase 1 is again applied in phase 2. Here, multiobjective GA is real coded having values from 0 to 1. Value ≥ 0.5 signifies the participation of base classifier in the ensemble and < 0.5 signifies nonparticipation concerned base classifiers in creating the ensembles. The output of phase 2 is an archive of the ensembles of the base classifiers in terms of chromosomes in the range of 0 and 1 (depicted in Figure 2). Here, value ≥ 0.5 signifies the participation of base classifier in ensemble and <0.5 signifies its non-participation. The set of ensembles provides the classification trade-offs for the user for different objective functions. Phase 3 of the proposed approach integrates the predictions of base classifiers to get a prediction of the final ensemble. As depicted in Figure 3, the phase takes two inputs: (1) archive of nondominated base solutions (output of phase 1); (2) one chromosome from the archive of ensembles as chosen by the user depending on requirements (output of Phase 2). The user may adopt static or a dynamic strategy to choose an appropriate ensemble from a pool of ensembles (evolved in Phase 2). Here in this work, we selected the ensemble classifier using a static strategy based on its performance on the training data in terms of predefined performance metrics. Based on the values of the chromosome, corresponding predictions of base classifiers are aggregated to get a final prediction of the ensemble. In order to test the proposed approach, test dataset is directly fed to different base classifiers. Their predictions are combined in this phase to give the final output of the ensemble. In this work, we computed the final prediction of ensemble by using the majority voting method because of its popularity as depicted in Figure 3.

The phases of the proposed approach address key issues of the current research in the field of ensembles. The issues addressed are (1) generation of a set of noninferior solutions that exhibit classification trade-offs to formulate base classifiers of the ensemble; (2) generation of a set of noninferior ensemble solutions that exhibit classification trade-offs; (3) integration of predictions of the base classifiers to get a final prediction of the ensemble.

### 3.2. Experiments

To evaluate the proposed approach, it is implemented in VC++. NB is used as a base classifier as per finding of state-of-the-art literature in the field of ID. The performance of the proposed approach is evaluated based on benchmark datasets for ID, namely, KDD cup 1999 and ISCX 2012 dataset. During the optimization of multiple criteria by AMGA2, the detection rate for each attack class in the dataset is used as a separate objective. The majority voting method is used to integrate the predictions of base classifiers to get a prediction of the final ensemble. The results of experiments are computed on a Windows PC with Core i3-2330M 2.20 GHz CPU and 2 GB RAM. The following subsections describe the brief review of genetic algorithms (GAs), naive bayes (NB), benchmark datasets, and performance metrics used in the experiments.

#### 3.2.1. Genetic Algorithm (GA)

GA is population based search technique that has been identified to perform better than the classical heuristics or gradient approaches [35]. GAs provides better solutions particularly for multimodels, nondifferentiable or discontinuous functions. Generally, GA experiences the following steps.

1. Generating a random population of individuals that represents a solution to the underlying problem.
2. Evaluating the population by computing the fitness function of each individual.
3. Elevating high quality individuals by selecting them from the entire population.
4. Generating new population containing individuals created by applying variation operators of crossover and mutation.
5. Repeating the above steps till termination criteria are satisfied.

A large number of methods have been developed to implement steps for GAs. However, major issues consist of representation of individuals, fitness evaluation mechanism, variation operators of crossover and mutation, and deciding the termination criteria.

#### 3.2.2. Naive Bayes (NB)

Bayes networks are one of the most widely used graphical models to represent and handle uncertain information [51, 52]. Generally, Bayes networks are described by two components: graphical component and numerical component.

- A graphical component is composed of a directed acyclic graph (DAG) where vertices represent events and edges are relations between events.
- A numerical component consists in a quantification of different links in the DAG by a conditional probability distribution of each node in the context of its parents.

Naive Bayes are simple Bayes networks which are composed of DAGs with only one root node (called parent) representing the unobserved node and several children, corresponding to observed nodes, with the strong assumption of independence among child nodes in the context of their parent. The classification is ensured by considering the parent node to be a hidden variable stating to which class each object in the testing set should belong and child nodes represent different attributes specifying this object. Hence, in the presence of a training set, only the conditional probabilities are computed since the structure is unique. Once, the network is quantified, it is possible to classify any new object giving its attribute values using Baye's rule. Baye's rule can be expressed as,

(1) $$\begin{array}{c} P\left(c_{i}\;|\;A\right)=\frac{P\left(A\;|\;c_{i}\right)\cdot P\left(c_{i}\right)}{P\left(A\right)}, \end{array}$$

where *c*_*i*_ is a possible value in the session class and *A* is the total evidence on attribute nodes. The evidence *A* can be dispatched into pieces of evidence, say *a*_1_, *a*_2_,…, *a*_*n*_, relative to the attributes *A*_1_, *A*_2_,…, *A*_*n*_, respectively. Since naive Bayes works under the assumption that these attributes are independent (giving the parent node *C*), their combined probability is obtained as follows:

(2) $$\begin{array}{c} P\left(c_{i}\;|\;A\right)=\frac{P\left(a_{1}\;|\;c_{i}\right)\cdot P\left(a_{2}\;|\;c_{i}\right)\cdots P\left(a_{n}\;|\;c_{i}\right)\cdot P\left(c_{i}\right)}{P\left(A\right)}. \end{array}$$

Note that there is no need to explicitly compute the denominator *P*(*A*) since it is determined by the normalization condition.

#### 3.2.3. Benchmark Datasets

The performance of the proposed approach is measured based on benchmark datasets. In the literature, various benchmark data sets are proposed for validation of the IDSs. As per statistics of a survey of 276 papers published between 2000 and 2008 conducted by Tavallaee [53], most of the researchers used publicly available benchmark datasets for evaluating their network based approaches. It is observed that KDD cup 1999 data set [54] is the most widely data set used for validation of an IDS [2, 53] in spite of many criticisms [4, 55, 56]. The raw training dataset contains about 4 GB of TCP connection data in the form of 5 million connection records. Similarly, test data set contains about 2 million records. KDD cup 1999 dataset utilizes TCP/IP level information and embedded with domain-specific heuristics to detect intrusions at the network level. KDD dataset contains four major classes of attacks: probe, denial of service (DoS), user-to-root (U2R), and remote-to-local (R2L) attacks. The labelled connection records consist of 41 features and 01 attack type. The labelled connection records consist of 22 different attack types categorized into 04 classes whereas unlabeled dataset consists of 20 known and 17 unknown attack types. The 41 features can be divided into three categories, namely, basic features of individual TCP connections, content features within a connection suggested by domain knowledge, and Traffic features computed using a two-second time window.

In a thorough study of KDD cup 1999 dataset, Tavallaee [53] observed that there are some inherent problems. He refined the KDD cup 1999 dataset and named it as NSL-KDD dataset. As the number of connection records in training and test data set is very large, so it is practically very difficult to use the whole data set. Thus, in order to conduct unbiased learning and testing of the proposed approach, we used subsets of the dataset containing different proportions of normal and attack instances. The statistics of selected subsets of NSL-KDD datasets used in our experiments are as depicted in Table 1.

In order to overcome the limitations of KDD cup 1999 dataset, Shiravi et al. [57] presented a new dataset for validation of an IDS at Information Security Center of excellence (ISCX). The dataset is available in the packet capture form. Features are extracted from the packet format by using tcptrace utility (downloaded from http://www.tcptrace.org) and applying the following command:

 tcptrace csv-l filename1.7z > filename1.csv,

where filename is the name of the 7z (packet capture) file. From resulting csv files, we selected features which are the most widely used features in the literature as proposed by Brugger [58]. The data instances including normal as well as attack instances are randomly selected to create a subset of the benchmark dataset for our experiments. The selected dataset is further preprocessed by converting discrete feature values to numeric ones as described in Kumar et al. [59]. The statistics of selected ISCX 2012 data subset are depicted in Table 2.

#### 3.2.4. Performance Metrics

In order to evaluate the effectiveness of the IDS, we measure its ability to correctly classify events as normal or intrusive along with other performance objectives, such as economy in resource usage, resilience to stress, and the ability to resist attacks directed at the IDS [60]. Measuring this ability of the IDS is important to both industry as well as the research community. It helps us to tune the IDS in a better way as well as compare different IDSs. There exist many metrics that measure different aspects of the IDS, but no single metric seems sufficient to objectively measure the capability of the IDS. Most widely used metrics by the intrusion detection research community are true positive rate (TPR) and false positive rate (FPR). Or false negative rate FNR = 1-TPR and true negative rate TNR = 1-FPR can also be used alternatively. Based upon values of these two metrics only, it is very difficult to determine best IDS among different IDSs. For example, one IDS reports that TPR = 0.8; FPR = 0.1, while at another IDS, TPR = 0.9; FPR = 0.2. If only values of TPR and FPR are given, then it is very difficult to determine the best IDS. To solve this problem, Gu et al. [60] proposed a new objective metric called intrusion detection capability (CID) considering base rate, TPR, and FPR collectively. CID possesses many important features. For example, (1) it naturally takes into account all the important aspects of detection capability, that is, FPR, FNR, positive predictive value (PPV) [19], negative predictive value (NPV), and base rate (the probability of intrusions); (2) it objectively provides an essential measure of intrusion detection capability; (3) it is very sensitive to IDS operation parameters such as base rate, FPR, and FNR. Details of CID can be further studied in Gu et al. [60]. Keeping these points in view, we computed TPR, FPR, and CID to evaluate the performance of the proposed technique and compare it with other representative techniques in the field.

#### 3.2.5. Experimental Setup

In this investigation, we used AMGA2 as a multiobjective genetic algorithm because of its benefits over other representative algorithms [48]. The implementation of AMGA2 algorithm takes the following input parameters:

- number of function evaluations;
- number of desired solutions;
- random seed;
- output file.

Rest of parameters like mutation rate, crossover rate, and so forth are automatically tuned by the AMGA2 algorithm.

The proposed approach involves three phases to create the ensemble as described in Section 3.1. In phase 1 (ensemble generation phase), AMGA2 optimizes an archive of a diverse set of feature subsets of datasets for predicting the target class. The optimized feature subsets are used to train NB classifiers which exhibit classification trade-offs for the user. The values in chromosome represent the features of the dataset to be given as input to NB classifiers. The size of chromosomes is equal to the number of features of a dataset under consideration. Each chromosome represents a subset of features of a dataset. The output of phase 1 is a set of optimized values indicating involvement of corresponding features in predicting target class using NB classifier. The NB classifiers trained using an optimized subset of features formulate the base classifiers for the ensembles. In phase 2 (ensemble selection phase), AMGA2 is again used to create an archive of the ensembles that also exhibit classification trade-offs. In phase 3 (ensemble integration phase), the predictions of selected base classifiers are combined to compute the final prediction of the ensemble using the majority voting method. The parameters used as input by the user to AMGA2 are depicted in Table 3. Other simulation parameters are tuned automatically by AMGA2 for KDD cup 1999 dataset and the ISCX 2012 dataset are presented in Tables 4 and 5, respectively.

### 3.3. Results

For investigation of NB as a base classifier, ensemble generation is done by using different subsets of the feature space (feature level). Diverse set of NB classifiers is generated by optimizing those using different subsets of the feature space of the training data set. In the ensemble selection phase, we selected the NB classifiers for the final ensemble based on their performance during the training process (overproduce-and-choose strategy). Finally, the ensemble integration phase involves fusion strategy (majority voting method) to combine the predictions of the selected classifiers.

In our experiments, we selected the solution for comparison with other classifiers having a better value of the CID. The results of the proposed intrusion detection technique using NB as a base classifier and other representative techniques are computed based upon benchmark datasets in terms of defined performance metrics. We computed average DR, average FPR, CID, and DR of each target class from confusion matrices. The representative techniques used in this investigation are naive Bayes and its ensembles using bagging and boosting. We utilized WEKA software package to compute the results of NB and its ensembles using bagging and boosting methods. We used default parameters of WEKA for computing the results using NB and its ensembles.

#### 3.3.1. Results of KDD Cup 1999 Dataset

**Figure 4:**
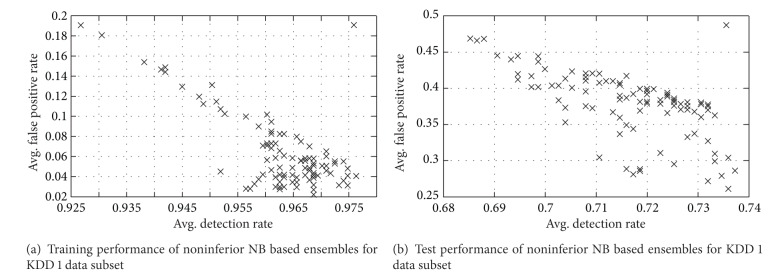
Training and test performance of noninferior NB based ensembles for KDD 1 data subset.

The proposed approach is applied to various data subsets of KDD cup 1999 dataset that produces a set of noninferior solutions using NB as base classifiers. The performance of solutions for training and test data of KDD 1 dataset is as depicted in Figure 4.

**Figure 5:**
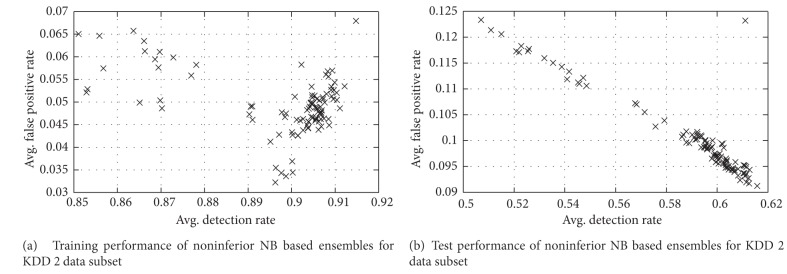
Training and test performance of noninferior NB based ensembles for KDD 2 data subset.

The performance of solutions for training and test data of KDD 2 dataset is as depicted in Figure 5.

**Figure 6:**
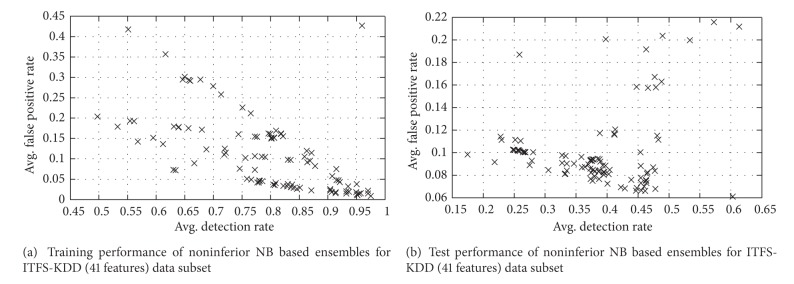
Training and test performance of noninferior NB based ensembles for ITFS-KDD (41 features) data subset.

The performance of solutions for training and test data of ITFS-KDD (41 features) dataset is described in Figure 6.

**Figure 7:**
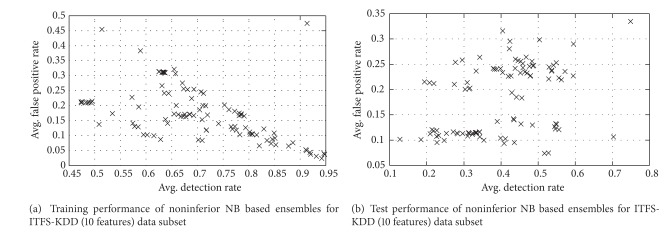
Training and test performance of noninferior NB based ensembles for ITFS-KDD (10 features) data subset.

The performance of solutions for training and test data of ITFS-KDD (10 features) dataset is described in Figure 7. The overview of classification results of KDD subsets obtained with NB and its ensembles (bagging and boosting methods) and the proposed approach (AMGA2-NB) with respect to different evaluation criteria is depicted in Table 6. The results indicate that NB and its ensembles using conventional techniques show comparable performance. These techniques produce better results for the majority attack classes. But, these techniques detect minority classes like U2R and R2L poorly. This proves that NB trained using conventional methods for bagging and boosting is more biased towards the majority attack classes. Whereas higher values of CID for AMGA2-NB indicate its better performance than other techniques considered in this investigation.

#### 3.3.2. Results of ISCX 2012 Dataset

**Figure 8:**
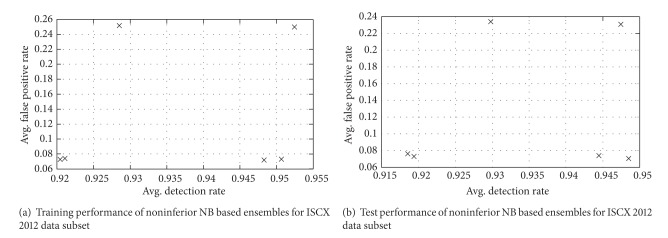
Training and test performance of noninferior NB based ensembles for ISCX 2012 data subset.

The performance of solutions for training and test data of ISCX 2012 dataset is described in Figure 8. The overview of detection results of ISCX 2012 subset obtained with NB and its ensembles and the proposed approach (ANGA2-NB) with respect to different evaluation criteria is as depicted in Table 7. It can be observed from the reporting results that AMGA2-NB (NB trained with the proposed technique) reported superior performance than NB and its ensembles based on bagging and boosting. AMGA2-NB reported the detection of normal and attack classes up to 95.2% and 92.7%, respectively. Higher value of CID indicates that our proposed technique outperformed the other techniques for the ISCX 2012 dataset considered in this investigation.

#### 3.3.3. Discussion

The results obtained in this study clearly highlight the benefits of trained NB and its ensembles by using the proposed technique. Higher values of CID corresponding to AMGA2-NB prove its superiority over the other techniques. The percentage improvement of the results of the proposed technique over the other techniques is also depicted in Table 8. The results indicate that the proposed technique helps to enhance the average detection rate, reduce average false positive rate, and increase CID values over that of other techniques. The higher values of CID for AMGA2-NB in comparison to the other techniques prove its superiority.

In a nutshell, the empirical investigation and comparison of the results indicate the following.

- The proposed approach outperforms the individual representative techniques in terms of identified performance metrics.
- There are indications in the literature that bagging and boosting learn better from imbalanced data. However, the experiments here have demonstrated that these algorithms remain biased towards the majority class(es).
- Using NB as a base classifier, the proposed approach is able to enhance DR by 35% and reduce FPR by 55% approximately over the results of NB technique and its ensemble using boosting technique based on KDD cup 1999 dataset as depicted in Table 8. However, an improvement of results is noticed up to 36% in DR and 55% in FPR approximately over bagging based ensemble of NB for KDD cup 1999 dataset. For ISCX 2012 dataset, the results of the proposed technique are improved up to 118% in DR and 24% in FPR approximately over NB and its ensemble using bagging technique.
- The ensembles evolved with the proposed technique provide better solutions and also achieve a higher detection accuracy.
- Higher values of CID for the proposed approach proved the superiority over the existing individual techniques and their ensembles using bagging and boosting.
- The proposed approach is capable of producing a pool of solutions that address the limitations of the existing techniques, striving to obtain a single solution in which there is no control on classification trade-offs (for application specific requirements).
- The proposed approach is a generalized classification approach that is applicable to the problem of any field having multiple conflicting objectives, and a dataset can be represented in the form of labelled instances in terms of its features.

## 4. Conclusion and Scope for Future Work

In this paper, a novel multiobjective optimization approach is proposed for effective intrusion detection. The proposed approach is capable of producing a pool of noninferior individual solutions and ensemble solutions thereof which exhibit classification trade-offs for the user. By using certain heuristics or prior domain knowledge, a user can select an ideal solution as per application specific requirements. The proposed approach attempts to tackle the issues of low DR, high FPR, and lack of classification trade-offs in the field of ID. The proposed approach consists of encoding of chromosomes that provides an optimized subset of features of a dataset. The optimized feature subset can be furthered to train a diverse set of NB classifiers that formulate base classifiers for ensembles. AMGA2 algorithm is employed to build multiobjective optimization model that generates an optimized subset of features with simultaneous consideration of detection rate of each attack class in the dataset. A three-phased multiobjective optimization approach can rapidly generate numerous individual solutions and ensemble solutions thereof with simple chromosome design in the first phase of the proposed approach. The entire solutions are further refined to obtain ensemble solutions in the second phase of the approach. The predictions of individual solutions are fused together to compute final prediction of the ensemble using the majority voting method in phase 3 of the proposed approach.

Benchmark datasets, namely, KDD cup 1999 and ISCX 2012 dataset for intrusion detection, are used to demonstrate and validate the performance of the proposed approach based on NB as a base classifier. The proposed approach can discover an optimized set of features that can be further used to train NB classifiers and ensemble of NBs thereof with a good support and a detection rate from benchmark datasets (in comparison with well-known ensemble methods like bagging and boosting). The optimized ensembles of NBs exhibit the classification tradeoffs for the users. The user may select an ideal solution as per application specific requirements.

The major issue in the proposed approach is that it takes long time to compute fitness functions in various generations. It may be overcome by computing the function values in parallel. Here, we computed the results by limiting the population size and number of generations of MOGA. More experiments may be conducted by using different values of these parameters. The proposed approach is validated using small subsets of benchmark datasets only, whereas its applicability can be tested by conducting more experiments with real network traffic in the field of ID. The proposed approach utilized static method for selecting an appropriate ensemble solution, whereas dynamic selection method may lead to more fruitful results.

## Figures and Tables

**Table 1:** Statistics of subsets of KDD cup 1999 dataset as training and test dataset.

Dataset	Mode	#F	Class	#I	Total
KDD 1	Training	41	Normal	1000	
			Probe	100	
			DoS	100	
			U2R	11	
			R2L	100	1311
	Test	41	Normal	500	
			Probe	75	
			DoS	75	
			U2R	50	
			R2L	50	750

KDD 2	Training	41	Normal	13449	
			Probe	2289	
			DoS	9234	
			U2R	11	
			R2L	209	25192
	Test	41	Normal	2152	
			Probe	2402	
			DoS	4342	
			U2R	200	
			R2L	2754	11850

ITFS KDD	Training	41, 10	Normal	10000	
			Probe	32316	
			DoS	23467	
			U2R	52	
			R2L	1126	66961
	Test	41, 10	Normal	5000	
			Probe	4166	
			DoS	17761	
			U2R	228	
			R2L	13448	40603

**Table 2:** Statistics of subset of ISCX 2012 dataset as training and test dataset.

Dataset	Mode	#F	Class	#I	Total
ISCX 2012	Training	9	Normal	4125	
			Attack	578	4703
	Test	9	Normal	64127	
			Attack	577	4704

**Table 3:** Parameters of AMGA2 input by the user.

Number of function evaluations	25000
Number of desired solutions	100
Random seed	0.1

**Table 4:** Simulation parameters tuned by AMGA2 for KDD cup 1999 dataset.

Parameter	Value
Maximum allowed size of archive	Number of desired solutions input by the user
Size of initial population	Number of desired solutions input by the user
Size of working population	20
Maximum number of function evaluations	Number of function evaluations input by the user
Probability of crossover	0.1
Probability of mutation	0.01
Index for crossover	0.5
Index for mutation	15

**Table 5:** Simulation parameters tuned by AMGA2 for ISCX 2012 dataset.

Parameter	Value
Maximum allowed size of archive	Number of desired solutions input by the user
Size of initial population	Number of desired solutions input by the user
Size of working population	8
Maximum number of function evaluations	Number of function evaluations input by the user
Probability of crossover	0.1
Probability of mutation	0.111111
Index for crossover	0.5
Index for mutation	15

**Table 6:** Overview of classification results of KDD cup 1999 subsets using NB as a base classifier.

Dataset	Technique	Avg. DR	Avg. FPR	CID	Normal	Probe	DoS	U2R	R2L
KDD 1	NB	0.619	0.208	0.129	0.698	0.960	0.387	0.140	0.140
	Bagged-NB	0.651	0.219	0.140	0.746	0.960	0.387	0.140	0.140
	Boosted-NB	0.619	0.208	0.129	0.698	0.960	0.387	0.140	0.140
	AMGA2-NB	0.736	0.260	**0.165**	0.872	0.893	0.413	0.140	0.220

KDD 2	NB	0.549	0.085	0.157	0.691	0.939	0.449	0.180	0.281
	Bagged-NB	0.549	0.085	0.157	0.691	0.939	0.449	0.180	0.281
	Boosted-NB	0.548	0.085	0.157	0.691	0.939	0.449	0.170	0.280
	AMGA2-NB	0.616	0.091	**0.194**	0.820	0.945	0.450	0.200	0.461

ITFS KDD 41 features	NB	0.446	0.120	0.074	0.945	0.972	0.353	0.254	0.223
	Bagged-NB	0.442	0.122	0.071	0.944	0.957	0.351	0.241	0.221
	Boosted-NB	0.446	0.120	0.074	0.945	0.972	0.353	0.254	0.223
	AMGA2-NB	0.604	0.060	**0.197**	0.855	0.997	0.287	0.145	0.814

ITFS KDD 10 features	NB	0.566	0.233	0.067	0.775	0.718	0.657	0.171	0.326
	Bagged-NB	0.540	0.237	0.056	0.775	0.717	0.599	0.158	0.326
	Boosted-NB	0.566	0.233	0.067	0.775	0.718	0.657	0.171	0.326
	AMGA2-NB	0.703	0.105	**0.226**	0.807	0.896	0.615	0.118	0.731

**Table 7:** Overview of comparative results of ISCX 2012 subsets using NB as a base classifier.

Dataset	Technique	Avg. DR	Avg. FPR	CID	Normal	Attack
ISCX 2012	NB	0.432	0.093	0.107	0.355	0.984
	Bagged-NB	0.453	0.090	0.121	0.378	0.984
	Boosted-NB	0.432	0.093	0.107	0.355	0.984
	AMGA2-NB	0.945	0.070	**0.593**	0.952	0.927

**Table 8:** Percentage improvement of the results of the proposed technique using NB as a base classifier.

Classifier	NB			Boosted	NB		Boosted	NB	
Dataset	DR	FPR	CID	DR	FPR	CID	DR	FPR	CID
KDD 1	18.90	25.00	27.91	13.06	18.72	17.86	18.90	25.00	27.91
KDD 2	12.20	7.06	23.57	0.12	0.07	0.24	12.20	7.06	23.57
ITFS 41	35.43	−50.00	166.22	36.65	−50.82	177.46	35.43	−50.00	166.22
ITFS 10	24.20	−54.94	237.31	30.19	−55.70	318.52	24.20	−54.94	237.31
ISCX 2012	118.75	−24.73	454.21	108.61	−22.22	390.08	118.75	−24.73	454.21
